# Long-term outcomes of polycythemia vera patients treated with ropeginterferon Alfa-2b

**DOI:** 10.1038/s41375-022-01528-x

**Published:** 2022-02-24

**Authors:** Jean-Jacques Kiladjian, Christoph Klade, Pencho Georgiev, Dorota Krochmalczyk, Liana Gercheva-Kyuchukova, Miklos Egyed, Petr Dulicek, Arpad Illes, Halyna Pylypenko, Lylia Sivcheva, Jiří Mayer, Vera Yablokova, Kurt Krejcy, Victoria Empson, Hans C. Hasselbalch, Robert Kralovics, Heinz Gisslinger, the PROUD-PV Study Group, Jean-Jacques Kiladjian, Pencho Georgiev, Dorota Krochmalczyk, Liana Gercheva-Kyuchukova, Miklos Egyed, Petr Dulicek, Arpad Illes, Halyna Pylypenko, Lylia Sivcheva, Jiří Mayer, Vera Yablokova, Heinz Gisslinger

**Affiliations:** 1 Université de Paris, AP-HP, Hôpital Saint-Louis, Centre d’Investigations Cliniques, INSERM, CIC1427 Paris, France; 2 AOP Orphan Pharmaceuticals, Vienna, Austria; 3 Medical University of Plovdiv, Plovdiv, Bulgaria; 4 Teaching Unit of the Hematology Department, University Hospital in Krakow, Krakow, Poland; 5 Clinical Hematology Clinic, Multiprofile Hospital for Active Treatment “Sveta Marina”, Varna, Bulgaria; 6 Department of Internal Medicine II, Kaposi Mor County Teaching Hospital, Kaposvar, Hungary; 7 Department of Clinical Hematology, University Hospital Hradec Kralove, Hradec Kralove, Czech Republic; 8 Department of Hematology, Faculty of Medicine, University of Debrecen, Debrecen, Hungary; 9 Department of Hematology, Regional Treatment and Diagnostics Hematology Centre, Cherkasy Regional Oncology Centre, Cherkasy, Ukraine; 10 First Department of Internal Medicine, Multiprofile Hospital for Active Treatment - HristoBotev, Vratsa, Bulgaria; 11 Clinic of Internal Medicine - Hematology and Oncology, University Hospital Brno, Brno, Czech Republic; 12 Department of Hematology, Yaroslavl Regional Clinical Hospital, Yaroslavl, Russia; 13 Department of Hematology, Zealand University Hospital, Roskilde, University of Copenhagen, Copenhagen, Denmark; 14 Department of Laboratory Medicine, Medical University of Vienna, Vienna, Austria; 15 CeMM Research Center for Molecular Medicine of the Austrian Academy of Sciences, Vienna, Austria; 16 Department of Internal Medicine I, Division of Hematology and Blood Coagulation, Medical University Vienna, Vienna, Austria; 17 Université de Paris, AP-HP, Hôpital Saint-Louis, Centre d’Investigations Cliniques, INSERM, CIC1427 Paris, France; 18 Medical University of Plovdiv, Plovdiv, Bulgaria; 19 Teaching Unit of the Hematology Department, University Hospital in Krakow, Krakow, Poland; 20 Clinical Hematology Clinic, Multiprofile Hospital for Active Treatment “Sveta Marina”, Varna, Bulgaria; 21 Department of Internal Medicine II, Kaposi Mor County Teaching Hospital, Kaposvar, Hungary; 22 Department of Clinical Hematology, University Hospital Hradec Kralove, Hradec Kralove, Czech Republic; 23 Department of Hematology, Faculty of Medicine, University of Debrecen, Debrecen, Hungary; 24 Department of Hematology, Regional Treatment and Diagnostics Hematology Centre, Cherkasy Regional Oncology Centre, Cherkasy, Ukraine; 25 First Department of Internal Medicine, Multiprofile Hospital for Active Treatment - HristoBotev, Vratsa, Bulgaria; 26 Clinic of Internal Medicine - Hematology and Oncology, University Hospital Brno, Brno, Czech Republic; 27 Department of Hematology, Yaroslavl Regional Clinical Hospital, Yaroslavl, Russia; 28 Department of Internal Medicine I, Division of Hematology and Blood Coagulation, Medical University Vienna, Vienna, Austria

**Keywords:** Drug development, Cancer immunotherapy

## To the Editor:

Interferon alfa not only restores normal blood cell counts in patients with polycythemia vera (PV) but can diminish the mutant *JAK2*V617F allele burden [1–3]. After discontinuing long-term interferon therapy, hematologic responses may persist [4, 5], which is more likely in patients achieving *JAK2*V617F allele burden <10% before stopping treatment [6]. Allele burden declines gradually during interferon treatment [7]; however, no data from large, prospective, clinical studies of long-term treatment with pegylated alfa interferons are available.

Results from 3 years’ treatment in the phase 3, open-label, randomized clinical trial PROUD-PV and its phase 3b extension trial CONTINUATION-PV [8] led to regulatory approval of ropeginterferon alfa-2b (BESREMi®), a novel, monopegylated interferon alfa-2b with an extended administration interval of 2–4 weeks, in the European Union. The compound was subsequently approved by the US FDA for first-line treatment of PV. Here we report hematologic and molecular responses and safety results after 5 years’ treatment in PROUD-PV and CONTINUATION-PV, which compared the efficacy and safety of ropeginterferon alfa-2b with hydroxyurea in the first year, and with best available treatment (BAT) thereafter.

Study design and methods were published previously [8]; selection criteria, dosing and endpoints are described in the supplement (Supplementary Tables 1–3 and Supplementary Fig. 1). *JAK2*V617F-positive patients with PV who were hydroxyurea naïve or hydroxyurea pre-treated for <3 years without complete response, resistance or intolerance were randomized 1:1 (stratified by age, history of thromboembolic events and hydroxyurea pretreatment) in PROUD-PV to receive ropeginterferon alfa-2b or hydroxyurea for 12 months. Dosing increased until blood counts normalized. Patients completing PROUD-PV were invited to enter CONTINUATION-PV: the ropeginterferon alfa-2b arm continued the same treatment with individualized dosing every 2, 3, or 4 weeks, and patients allocated to hydroxyurea in PROUD-PV received BAT (hydroxyurea or another standard first-line treatment with individualized dosing) in CONTINUATION-PV.

Assessment visits were performed 3–6 monthly to determine efficacy (*JAK2*V617F allelic burden [*JAK2*V617F ipsogen® JAK2 MutaQuant® kit, QIAGEN GmbH, Hilden, Germany; limit of background: 0.014%], hematocrit, platelet, leukocyte and erythrocyte counts, phlebotomy requirement, spleen size, and quality of life) and safety parameters (including clinical chemistry, immunological parameters, urinalysis, Hospital Anxiety and Depression Scale score and adverse events).

The studies were conducted in accordance with the Declaration of Helsinki, received ethics committee approval, and were registered at www.clinicaltrials.gov (#NCT01949805; #NCT02218047). All patients gave informed consent.

Efficacy data up to Month 60 were analyzed in patients who entered CONTINUATION-PV (*N* = 171; full analysis set, treatment as assigned). Safety data up to database lock (29 May 2020) were analyzed for all patients treated (*N* = 254). Efficacy between treatment groups was compared using a log binomial regression model. Rate ratios (RR) of responders between arms and 95% CI were calculated from estimates of regression coefficients. Last observation carried forward was imputed for molecular parameters. Safety was analyzed descriptively. Statistical Analysis System® software was used (version 9.3 or higher, SAS Institute, Cary, NC, USA).

Patient characteristics and disposition are reported in Supplementary Table 4 and Supplementary Fig. 2. At database lock, 70/95 patients in the ropeginterferon alfa-2b arm and 57/76 in the BAT arm remained in the extension study, while 25/95 (26.3%) and 19/76 (25.0%), respectively had discontinued. Most patients in the BAT arm received only hydroxyurea, but at 60 months, 5 had switched to interferon therapy (ropeginterferon alfa-2b, pegylated interferon alfa-2a, or recombinant interferon alfa-2a) and 2 had switched to ruxolitinib.

After 5 years’ treatment, 53/95 patients (55.8%) in the ropeginterferon alfa-2b arm and 33/75 (44.0%) in the control arm achieved complete hematologic response (CHR) according to modified European LeukemiaNet (ELN) criteria [9] (RR: 1.30 [95% CI: 0.95 to 1.77]; *p* = 0.0974; discontinued patients were considered non-responders). Response rates in a prospectively planned sensitivity analysis of CHR with imputation of the last observation carried forward were 69/95 (72.6%) in the ropeginterferon alfa-2b arm versus 40/76 (52.6%) in the control arm at Month 60 (RR: 1.43 [95% CI: 1.12–1.81]; *p* = 0.004).

**Fig. 1:**
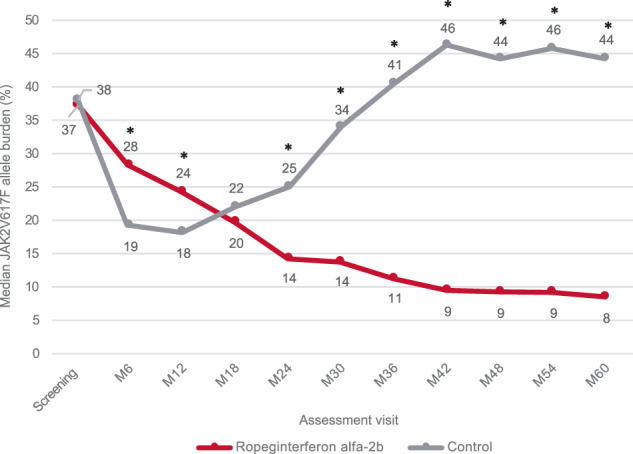
Median *JAK2*V617F allele burden (%). Figure 1 shows the median *JAK2*V617F alle burden (%) during a period of 5 years of treatment. *JAK2*V617F allele burden was determined every 6 months. Medians were calculated for the last observation carried forward at each timepoint assessed. * Denotes a statistically significant (p < 0.05) difference between the treatment arms.

The molecular response rate at 5 years according to ELN criteria [9] was significantly higher in the ropeginterferon alfa-2b arm than in the control arm (65/94 [69.1%] versus 16/74 [21.6%], respectively; RR: 3.04 [95% CI: 1.96–4.71]; *p* < 0.0001). The median *JAK2*V617F allele burden declined continuously during ropeginterferon alfa-2b treatment, from 37.3% at baseline (before treatment in PROUD-PV) to 8.5% at 60 months (Fig. 1). In contrast, the median *JAK2*V617F allele burden in the control arm decreased from 38.1% at baseline to 18.2% at 12 months but rebounded to 44.4% by 60 months (comparison of treatment arms: *p* < 0.0001). *JAK2*V617F allele burden <10%, achieved in 50/92 (54.3%) ropeginterferon alfa-2b-treated patients at Month 60 (Supplementary Table 5), correlated with lower age and lower *JAK2*V617F allele burden at baseline (Supplementary Table 6) and was associated with a higher complete hematologic response rate at Month 60 (Supplementary Table 7). Notably, allele burden decreased to <1% in 18/92 patients (19.6%) receiving ropeginterferon alfa-2b whereas in the control arm, only 1 patient (1.4%) achieved an allele burden <1% at 60 months (*p* = 0.0002). Mutant *JAK2*V617F allele burden routinely assessed in peripheral blood yields only a reflection of the effect in the bone marrow. Ropeginterferon alfa-2b has been shown to target the malignant cancer stem cell clone in patients [2]; its sustained effect on allele burden aligns with the hypothesis of gradual clonal exhaustion [10].

Disease progression (secondary myelofibrosis or leukemic transformation) and major thromboembolic events were assessed over a cumulative exposure period of 499 and 401 patient years in the ropeginterferon alfa-2b and control arms, respectively. The incidence of disease progression among ropeginterferon alfa-2b treated patients was 0.2%-patient years (1 case of myelofibrosis) versus 1.0%-patient years in the control treatment arm (2 cases of myelofibrosis and 2 cases of acute leukemia). Regarding major thromboembolic events, 5 events in 4 patients in the ropeginterferon alfa-2b arm and 5 events in 5 patients in the control arm occurred, incidence rates of 1.0%-patient year and 1.2%-patient year respectively. Thromboembolic events did not appear to correlate with higher hematocrit levels or greater phlebotomy need. All affected patients were aged ≥60 years and thus considered at high risk; no marked difference between the treatment arms was observed regarding other cardiovascular risk factors.

Rates of adverse events, serious adverse events and treatment-related adverse events over the entire treatment period were balanced between the study arms (Table 1). The most common adverse events (in > 10% of patients) in the ropeginterferon alfa-2b arm regardless of causality were thrombocytopenia, anemia, leukopenia, elevated hepatic enzymes, arthralgia, fatigue, headache, dizziness, splenomegaly, pyrexia, and back pain. In control-treated patients the most frequent adverse events were thrombocytopenia, anemia, leukopenia, fatigue, headache, nausea, diarrhea, influenza, and nasopharyngitis. Most adverse events were of grade 1–2 intensity. Treatment-related adverse events of grade ≥3 occurred in 21/127 patients (16.5%) in each arm; these included only 1 grade 4 event (increased gamma-glutamyltransferase in the ropeginterferon-alfa-2b arm) and 1 grade 5 event (acute leukemia with fatal outcome in the control arm). In total, 13/127 patients (10.2%) in the ropeginterferon-alfa-2b arm and 4/127 (3.1%) in the control arm discontinued treatment due to drug-related adverse events, approximately half of which occurred during the first year (in 6/13 and 2/4 patients, respectively).

**Table 1:** Summary of all adverse events in PROUD-PV and CONTINUATION-PV (Safety set).

		Ropeginterferon alfa-2b		Control	
		(499 patient years)		(401 patient years)	
Type of event	Intensity grade	Adverse events	Number of patients^a^	Adverse events	Number of patients^a^
			*n* (%)		*n* (%)
			*N* = 127		*N* = 127
Adverse events		1767	116 (91.3%)	1267	117 (92.1%)
Serious adverse events		56	30 (23.6%)	56	32 (25.2%)
Treatment-related serious adverse events		4	4 (3.1%)	7	5 (3.9%)
Adverse events related to polycythemia vera		171	48 (37.8%)	135	48 (37.8%)
Treatment-related adverse events		758	100 (78.7%)	458	100 (78.7%)
Intensity of treatment-related adverse events^b^	Grade 1	477	73 (57.5%)	277	79 (62.2%)
	Grade 2	241	75 (59.1%)	152	62 (48.8%)
	Grade 3	39	21 (16.5%)	28	20 (15.7%)
	Grade 4	1	1 (0.8%)	0	0
	Grade 5	0	0	1	1 (0.8%)
	Grade ≥3	40	21 (16.5%)	29	21 (16.5%)
Treatment-related adverse events leading to discontinuation		15	13 (10.2%)	4	4 (3.1 %)
Disease progression (myelofibrosis and leukemic transformation)^c^		1	1 (0.8%)	4	4 (3.1%)
Any neoplasm		16	12 (9.4%)	18	15 (11.8%)
Skin cancers related to treatment (basal cell carcinoma and malignant melanoma)		0	0	3	3 (2.4%)
Major thromboembolic adverse events^d^		5	4 (3.1%)	5	5 (3.9 %)

^a^Number of patients in whom the event was reported throughout the entire study period.

^b^Intensity grading according to CTCAE 4.0.

^c^One patient in the ropeginterferon alfa-2b arm developed myelofibrosis; in the control arm, 2 patients developed myelofibrosis and leukemic transformation occurred in 2 patients.

^d^Ropeginterferon alfa-2b arm: splenic infarction/truncus coeliacus thrombosis, intracardiac thrombus, hemorrhagic transformation stroke, and 2 cases of ischemic stroke. Control arm: embolism, femoral artery occlusion, superficial thrombophlebitis, venous thrombosis of the limb, and cerebrovascular accident.

Overall, results of the present analysis over ≥5 years’ treatment further indicate that ropeginterferon alfa-2b is an effective and safe option for longer-term treatment that induces a sustained molecular response, confirming data from an earlier phase I/II study also using ropeginterferon alfa-2b [11]. The majority of ropeginterferon alfa-2b-treated patients (54.3%) achieved a *JAK2*V617F allele burden <10% at 5 years and might be potential candidates for treatment discontinuation. Applying more stringent criteria (allele burden <10%, sustained complete hematologic response [hematocrit < 45% without phlebotomy in the last 3 months, platelet count <400 × 10^9^/L and leukocyte count <10 × 10^9^/L; drop-outs considered non-responders] for ≥2 years, and no disease progression, thromboembolic events, or worsening of disease-related signs or symptoms over the entire treatment period), 30.4% of patients who received ropeginterferon alfa-2b (versus 4.2% in the control arm; *p* < 0.0001) might be considered for treatment discontinuation according to previous findings [5, 6]. However, prerequisites for treatment stop remain a subject of research.

Disease transformation of *JAK2*V617F-positive MPNs may arise from accumulating genomic instability promoted by the expanding pool of homozygous *JAK2*V617F clones [12]. High *JAK2*V617F allele burden is a risk factor for progression to secondary myelofibrosis in patients with PV [13, 14]. Since alfa interferons reduce the *JAK2*V617F allele burden, this finding is congruent with an improved myelofibrosis-free survival rate reported among interferon-treated patients with PV compared to those receiving hydroxyurea or phlebotomy in a long-term retrospective study [15]. In the PROUD-PV/CONTINUATION-PV trials, conducted in an early-stage PV population, a fivefold lower incidence rate of disease progression (including myelofibrosis and leukemic transformation) was observed in ropeginterferon alfa-2b treated patients compared with the control arm, although these events were too rare to allow statistical comparison, which would require longer follow-up.

These new results provide further evidence for the possible disease modifying capability of ropeginterferon alfa-2b, which may alter the natural course of PV. In a sizable proportion of ropeginterferon alfa-2b-treated patients, treatment discontinuation (a goal that cannot be achieved in PV with any other currently available therapy) could potentially be considered, and warrants further study.

## Supplementary information

Supplemental data
