# Supplementary material for: Long-term outcomes of polycythemia vera patients treated with ropeginterferon Alfa-2b
Source: Leukemia. 2022 Feb 24;36(5):1408–11. doi: 10.1038/s41375-022-01528-x (PMC9061291; doi:10.1038/s41375-022-01528-x)
Supplement: Supplementary file 1 — Supplemental data [file 41375_2022_1528_MOESM1_ESM.docx]

# Supplement

Supplement to: Long-Term Follow Up of Patients Assigned to Ropeginterferon Alfa‑2b in the Randomized Phase 3 PROUD-PV trial and the Phase 3b CONTINUATION-PV Trial

Table of Contents

[Supplement 1](#_Toc90809638)

[Methods 2](#_Toc90809639)

[Selection of Study Population (PROUD-PV) 2](#_Toc90809640)

[Selection of Study Population (CONTINUATION-PV) 4](#_Toc90809641)

[Dosing Schemes 5](#_Toc90809642)

[Primary and Secondary Endpoints 7](#_Toc90809643)

[Safety Assessments 8](#_Toc90809644)

[Objectives 8](#_Toc90809645)

[Supplemental Statistical Methods 9](#_Toc90809646)

[Important changes to methods after trial commencement 10](#_Toc90809647)

[Supplementary results 11](#_Toc90809648)

[Dosing 11](#_Toc90809649)

[Tables 11](#_Toc90809650)

[Patient characteristics 11](#_Toc90809651)

[JAK2V617F allele burden 12](#_Toc90809652)

[Association of JAK2V617F allele burden of <10% at 5 years with baseline factors and other parameters at Month 60 12](#_Toc90809653)

[Figures 16](#_Toc90809654)

[Patient disposition 16](#_Toc90809655)

[Members of the PROUD-PV Study Group 18](#_Toc90809656)

[Institutional review board/ independent ethics committee approval 19](#_Toc90809657)

[References 20](#_Toc90809658)

## Methods

Methods of the PROUD-PV and CONTINUATION-PV have been published previously.^1^

***Setting and Dates***

The studies were conducted at 48 clinics in Europe. Recruitment took place from Sept 17, 2013 to March 13, 2015. Database lock for the 60-month analysis was on 29 May 2020.

### Selection of Study Population (PROUD-PV)

*Inclusion Criteria*

A patient who met all of the following criteria qualified for entry into the study:

- Male or female, 18 years or older.
- Diagnosis of polycythemia vera according to the World Health Organization (WHO) 2008 criteria^2^ with the mandatory presence of *JAK2*V617F mutation as the major disease criterion.
- For previously cytoreduction untreated patients – documented need for cytoreductive treatment (one or more of the following criteria):
  - Age > 60 years at the planned day of the first drug administration;
  - At least one previous well documented major cardiovascular polycythemia vera-related event, except bleeding and polycythemia vera-related thromboembolic complications in the abdominal area, see exclusion criterion 7) in the medical history;
  - Poor tolerance (defined as a phlebotomy/procedure-related adverse event [AE] causing significant adverse impact on the patient and limiting ability to apply phlebotomy with the intention to keep hematocrit < 45%) or frequent need for phlebotomy (more than one phlebotomy within last three months prior entering the study, while each of these phlebotomies was performed to reduce hematocrit level from > 45%, or if one phlebotomy was not able to reduce hematocrit level to < 45% for the next three months following phlebotomy);
  - Progressive splenomegaly (*de novo* appearance of a palpable spleen, or appearance of the symptoms, related to the enlarged spleen, e.g. pain, early satiety etc., with confirmed size increase);
  - Platelet count > 1000 x 10^9^/L (for two measurements within one week);
  - Leukocytosis (white blood cell count > 10 x 10^9^/L for two measurements within one week).
- For patients currently treated or pre-treated with hydroxyurea, all of the following criteria:
  - being non-responders (as defined by the response criteria for primary endpoint);
  - Total hydroxyurea treatment duration shorter than three years;
  - No documented resistance or intolerance as defined by modified criteria.^3^
- Hospital anxiety and depression scale (HADS) score 0-7 on both subscales.
- Patients with a HADS score of 8-10 inclusive on either or both of the subscales may have been eligible following psychiatric assessment that excluded clinical significance of the observed symptoms in the context of potential treatment with an interferon-α.
- Signed written informed consent.

*Exclusion Criteria*

A patient who met any of the following criteria did not qualify for entry into this trial:

- Any systemic cytoreduction for polycythemia vera in the medical history prior to study entry with exception of hydroxyurea for shorter than 3 years (see respective inclusion criterion).
- Any contraindication to any of the investigational medicinal products (pegylated interferon or hydroxyurea) or their excipients.
- Any systemic exposure to a non-pegylated or pegylated interferon-α in the medical history.
- Documented autoimmune disease at screening or in the medical history.
- Clinically relevant pulmonary infiltrates, pneumonia, and pneumonitis at screening.
- Infections with systemic manifestations, e.g., hepatitis B, hepatitis C, or human immunodeficiency virus (HIV) at screening.
- Known, polycythemia vera-related thromboembolic complications in the abdominal area (e.g. portal vein thrombosis, Budd-Chiari syndrome) and/or splenectomy in the medical history.
- Any investigational drug less than 6 weeks prior to the first dose of study drug or not recovered from effects of prior administration of any investigational agent.
- History or presence of depression requiring treatment with antidepressant.
- HADS score equal to or above 11 on either or both of the subscales.
- Any risk of suicide at screening or previous suicide attempts.
- Any significant morbidity or abnormality which may interfere with the study participation.
- Pregnancy and breast-feeding females of reproductive potential and males not using effective means of contraception. Note: women of childbearing potential not using effective contraceptive methods were not eligible for the study. A woman of childbearing potential was defined as any female having experienced menarche and who is not postmenopausal or permanently sterilized (e.g. tubal occlusion, hysterectomy, bilateral salpingectomy).
- History of active substance or alcohol abuse within the last year.
- Evidence of severe retinopathy (e.g. cytomegalovirus retinitis, macular degeneration) or clinically relevant ophthalmological disorder (due to diabetes mellitus or hypertension).
- Thyroid dysfunction (clinical symptoms of thyroid hyper- or hypofunction) not adequately controlled.
- Patients tested positively to thyroglobulin (TgAb) autoantibodies and / or thyroid peroxidase (TPOAb) autoantibodies at screening.
- History of major organ transplantation.
- History of uncontrolled severe seizure disorder.
- Leukocytopenia at the time of screening (leukocytes below the lower limit of normal).
- Thrombocytopenia at the time of screening (platelets below the lower limit of normal).
- History of malignant disease, including solid tumors and hematological malignancies (except basal cell and squamous cell carcinomas of the skin and carcinoma *in situ* of the cervix that have been completely excised and are considered cured) within the last 3 years.

With regard to aspirin (acetylsalicylic acid), the following contraindications are known for low dose acetylsalicylic acid: active peptic ulceration or history of peptic ulceration, hemophilia, hypersensitivity to acetylsalicylic acid or any other non-steroidal anti-inflammatory drugs (NSAIDs), including those in whom attacks of asthma, angioedema, urticaria, rhinitis have been precipitated by acetylsalicylic acid or any other NSAID, hypersensitivity to any of the other constituents. If any of the contraindications were observed, the patient was still allowed to participate in the study without being administered aspirin (acetylsalicylic acid).

### Selection of Study Population (CONTINUATION-PV)

*Inclusion Criteria*

A patient who met all of the following criteria qualified for entry into CONTINUATION-PV:

1. Patients who completed the PROUD-PV trial with:

a. Normalization of at least two out of three main blood parameters (hematocrit, platelets and white blood cells) if these parameters were moderately increased (hematocrit < 50%, white blood cell count < 20 x 10^9^/L, platelet count < 600 x 10^9^/L) at baseline of the PROUD-PV trial, OR

b. Greater than 35% decrease of at least two out of three main blood parameters (hematocrit, platelets and white blood cells) if these parameters were massively increased (hematocrit > 50%, white blood cell count > 20 x 10^9^/L, platelet count > 600 x 10^9^/L), at baseline of the PROUD-PV trial, OR

c. Normalization of spleen size, if spleen was enlarged at baseline of the PROUD-PV trial, OR

d. Otherwise a clear, medically verified benefit from treatment with ropeginterferon alfa-2b (e.g. normalization of disease-related micro-vasculatory symptoms, substantial decrease of *JAK2*V617F allelic burden).

2. Signed written informed consent form.

*Exclusion Criteria*

Patients who met any of the following criteria did not qualify for entry into CONTINUATION-PV:

Withdrawal criteria, as specified in the predecessor trial PROUD-PV, which mandated treatment discontinuation.

1. Non-recovery from the ropeginterferon alfa-2b related toxicities to the grade (usually, grade I) which allowed continuation of the treatment.

2. Hospital Anxiety and Depression Scale depression scale score of 11 or higher on either or both of the subscales, and /or development or worsening of the clinically significant depression or suicidal thoughts.

3. Progressive and clinically significant increase of liver enzyme levels despite dose reduction, or if such increase was accompanied by increased bilirubin level or any signs or symptoms of a clinically significant autoimmune disease.

4. Clinically significant development of a new ophthalmologic disorder, or worsening of a pre-existing one, during the study.

5. Ropeginterferon alfa-2b only: Loss of efficacy of ropeginterferon alfa-2b or any comparable situation where no further benefits of treatment continuation were expected by the Investigator.

### Dosing Schemes

PROUD-PV

During the initial treatment phase (first 12 weeks following randomization) in PROUD-PV, the dose was adjusted to achieve the optimal disease response of hematocrit < 45% without phlebotomies, platelet count of < 400 x 10^9^/L and leukocyte count of < 10 x 10^9^/L. If disease response was achieved, respective dose was continued in the maintenance phase (from week 13 onwards) of the study. Evaluation for the necessity of a dose modification was done at every treatment site visit.

*Ropeginterferon alfa-2b administration*

The dose escalation scheme during the initial treatment phase is presented in Supplemental Supplemental Table 1. Dose level 1 (50 µg) was the starting dose if patients switched from hydroxyurea; dose level 2 (100 µg) was the starting dose for cytoreductive naïve patients. Dose levels were escalated every two weeks until optimal disease response was achieved. The highest dose administered was dose level 10 (500 µg); hence the highest dose of ropeginterferon alfa-2b administered in the study was 500 µg every two weeks.

Supplemental Table 1: Dose Levels of Ropeginterferon alfa-2b in PROUD-PV and CONTINUATION-PV

|  | **PROUD-PV** | **CONTINUATION-PV** |
| --- | --- | --- |
| **Dose level** | **ropeginterferon alfa-2b dose, s.c. every two weeks** | **ropeginterferon alfa-2b, s.c. every 2, 3 or 4 weeks** |
| 1 | 50 µg* | 50 µg |
| 2 | 100 µg** | 100 µg |
| 3 | 150 µg | 150 µg |
| 4 | 200 µg | 200 µg |
| 5 | 250 µg | 250 µg |
| 6 | 300 µg | 300 µg |
| 7 | 350 µg | 350 µg |
| 8 | 400 µg | 400 µg |
| 9 | 450 µg | 450 µg |
| 10 | 500 µg | 500 µg |

* Starting dose if patients switched from hydroxyurea treatment.

** Starting dose for ropeginterferon alfa-2b cytoreductive naïve patients.

Abbreviations: s.c., subcutaneous.

*Hydroxyurea administration*

The dose escalation scheme for hydroxyurea during the initial treatment phase is presented in Supplemental Table 2. The daily dose was split into a morning and evening dose (the capsules were not broken). Dose level 2 (500 mg) was the starting dose.

Supplemental Table 2: Dose Levels of Hydroxyurea in PROUD-PV

| **Dose**  **level** | **Dose** **of** **hydroxyurea** |
| --- | --- |
| 1 | 500 mg every other day |
| 2 | 500 mg daily - starting dose |
| 3 | 500 mg day 1, 1000 mg day 2 - alternating |
| 4 | 1000 mg daily |
| 5 | 1000 mg day 1, 1500 mg day 2 - alternating |
| 6 | 1500 mg daily |
| 7 | 1500 mg day 1, 2000 mg day 2 - alternating |
| 8 | 2000 mg daily |
| 9 | 2500 mg daily |
| 10 | 3000 mg daily |

*Switching strategy for hydroxyurea treated patients assigned to the ropeginterferon alfa-2b treatment arm in PROUD-PV*

The PROUD-PV study protocol allowed enrolment of cytoreductive untreated polycythemia vera patients (naïve) and currently treated or pre-treated hydroxyurea patients (with a total hydroxyurea treatment duration < 3 years). For currently treated or pre-treated hydroxyurea patients, randomized to the ropeginterferon alfa-2b treatment arm, a transition phase was defined as initial part of the dose titration phase. Within a specified 12-week time period, the transition of patients with hydroxyurea treatment at the time of screening to ropeginterferon alfa-2b treatment was done. The treatment transition plan for these patients is summarized below and is outlined in Supplemental Figure 1.

Week 1 and Week 2: Patients received the same hydroxyurea dose as recorded at screening and received in addition 50 µg ropeginterferon alfa-2b (as a single subcutaneous application).

Week 3 to Week 12: Every two weeks, the hydroxyurea level was decreased to the next lower level. According to the titration dose scheme for hydroxyurea in the PROUD-PV Study, the dose level intervals were determined in 500 mg steps; if no lower dose level was available, hydroxyurea was discontinued. In parallel, every two weeks, the ropeginterferon alfa-2b dose level was increased to the next higher level according to the titration scheme for ropeginterferon alfa-2b in the study (i.e. the dose level intervals were determined in 50 µg steps); until (1) the individual, (disease response) maintenance dose was achieved, or (2) if no further dose level was available, the dose remained in the highest dose level (i.e. 500 µg ropeginterferon alfa-2b).

Week 13 onwards: Latest timepoint for discontinuation of the hydroxyurea weekly dose and further ropeginterferon alfa-2b administration at the maintenance dose.

Supplemental Figure 1: Switching Strategy for Hydroxyurea Treated Patients Assigned to the Ropeginterferon alfa-2b Treatment Arm in PROUD-PV

**
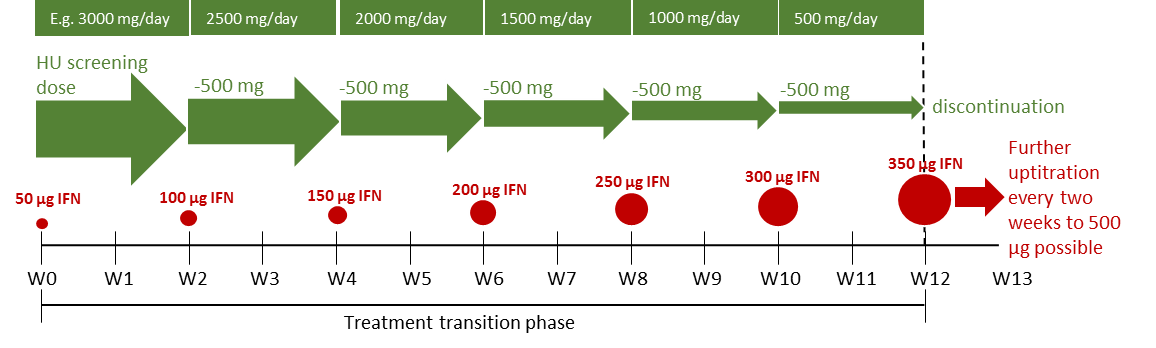
**

Abbreviations: hydroxyurea, hydroxyurea; W, week; IFN, ropeginterferon alfa-2b.

CONTINUATION-PV

The ropeginterferon alfa-2b dose achieving optimal disease response was determined according to PROUD-PV and continued in the extension study; dose adjustment at the investigator’s discretion was permitted. The BAT arm received hydroxyurea or another standard first line treatment for PV. Dosing in the BAT arm was at the investigator’s discretion based on disease response.

### Primary and Secondary Endpoints

The primary endpoints and main secondary endpoints of PROUD-PV and CONTINUATION-PV are presented in Supplemental Table 3.

Supplemental Table 3: Primary Endpoints and Main Secondary Endpoints of PROUD-PV and CONTINUATION-PV

| **PROUD-PV** | **CONTINUATION-PV** |
| --- | --- |
| *Primary endpoint*  Disease response rate at month 12:  Complete hematological response   - Hematocrit < 45% without phlebotomy (at least 3 months since last phlebotomy), - Platelet count < 400 x 10^9^/L, - Leukocyte count < 10 x 10^9^/L, and   Normal spleen size (≤ 12 cm females, ≤ 13 cm males) | *Co-primary endpoint*  Disease response rate at assessment visits (discontinued patient counted as non-responders):   - Complete hematological response - Hematocrit < 45% without phlebotomy (at least 3 months since last phlebotomy), - Platelet count < 400 x 10^9^/L, - Leukocyte count < 10 x 10^9^/L, and - Normal spleen size (≤ 12 cm females, ≤ 13 cm males);   and   - Complete hematological response and resolution and/or clinically improvement of disease-related signs and disease-related symptoms*   *Primary endpoint sensitivity analysis*   - Complete hematological response - Complete hematological response and splenomegaly defined as spleen size > 17 cm - Analysis of response rates with imputation of last observation carried forward for missing data due to missed assessments or discontinuation |
| *Main secondary endpoints*   - Complete hematological response - Change in hematological parameters from baseline - Change in spleen size from baseline - Time to disease response - Disease response duration - Phlebotomy need - Change of disease-related symptoms   *Other*   - Change in QoL (EQ-5D-3L) from baseline - Change in *JAK2*V617F allelic burden^§^ from baseline | *Main secondary endpoints*   - Change in hematological parameters from baseline - Change in spleen size from baseline - Time to disease response - Maintenance rate of disease response - Duration of response maintenance - Progression free time - Phlebotomy need - Change of disease related signs and disease-related symptoms - Change in QoL (EQ-5D-3L) from baseline - Change in *JAK2*V617F allelic burden^§^ from baseline |
| *Safety endpoints*   - Incidence, causality and intensity of AEs according to CTCAE 4.0. - Events leading to dose reduction or permanent treatment discontinuation - Adverse events of special interest | *Safety endpoints*   - Incidence, causality and intensity of AEs according to CTCAE 4.0. - Events leading to dose reduction or permanent treatment discontinuation - Adverse events of special interest** |

*Disease-related signs as assessed by the investigators was clinically significant splenomegaly. Disease-related symptoms as assessed by investigators included microvascular disturbances such as erythromelalgia, coronary artery disease, atypical TIAs, peripheral gangrene, amaurosis fugax, hemorrhages including bruises, ecchymoses, epistaxis bleeding after tooth extraction and gastrointestinal bleeding etc. as defined according to Michiels^4^, and pruritus and headache. Disease-related signs and symptoms were identified in medical history records and adverse events records. The occurrence of disease related signs and symptoms were analyzed descriptively by the same way as adverse events. Resolution and/or clinical improvement of disease-related signs and symptoms were binary variables (achieved, not achieved) and the same statistical methods of analysis as for the primary endpoint was used.

** Adverse events of special interest included psychiatric events (particularly depression associated with suicidal thoughts), ocular events (particularly events suspected to be due to thrombosis of ocular vessels), immunologic reactions including development of anti-thyroid antibodies with clinical symptoms as well as interferon-induced hypersensitivity and major disease-related cardiovascular events.

^§^ Measured using the *JAK2*V617F ipsogen® JAK2 MutaQuant® kit, QIAGEN GmbH, Hilden, Germany. Limit of detection: 0.061% (upper bound of 90% confidence interval: 0.091%). Values ≥ 0.091% were interpreted to indicate the presence of a *JAK2*V617F mutation. Limit of background: 0.014%; values ≤0.014% were considered undetectable.

### Safety Assessments

The main safety assessment included:

- Incidence, causality and intensity of AEs according to Common Terminology Criteria for Adverse Events (CTCAE) 4.0
- Laboratory safety data:
  - Hematology parameters: hemoglobin, platelet count, red blood cell count, white blood cell count with differential (neutrophils, lymphocytes, monocytes, eosinophils, basophils and reticulocytes), mean corpuscular volume
  - Blood chemistry: Blood urea nitrogen [BUN] or urea, alanine aminotransferase (ALT), aspartate aminotransferase (AST), gamma-glutamyl transferase (GGT), lactate dehydrogenase (LDH), total bilirubin, Na+, K+, Ca++, Cl-, uric acid, blood glucose, alkaline phosphatase, amylase, lipase, creatinine, total protein, albumin, cholesterol, triglycerides, serum iron, transferrin, thyroid stimulating hormone (TSH) and fT4
  - Coagulation parameters: Partial thromboplastin time (PTT) or activated partial thromboplastin time (aPTT), and fibrinogen
  - Immunological parameters: antinuclear antibodies (ANA), TgAb, TPOAb
- Standard 12-lead electrocardiogram (ECG)
- Physical examination
- Vital signs: heart rate, systolic and diastolic blood pressure
- Immunogenicity
- Urine, β2 microglobulin

All safety parameters were analyzed descriptively. All treatment-emergent adverse events (TEAE) were listed with Medical Dictionary for Regulatory Activities (MedDRA) coded terms. Separate listings were created for serious adverse events (SAEs), death and events leading to study discontinuation. Absolute and relative frequencies of patients with TEAEs and incidence of events by MedDRA primary System Organ Class (SOC) and preferred term (PT) were calculated.

### Objectives

PROUD-PV

Primary objective:

To demonstrate non-inferiority of ropeginterferon alfa-2b vs. HU in terms of disease response rate in both HU naïve and currently treated patients, diagnosed with polycythemia vera.

Secondary objectives:

Efficacy in the two treatment arms, safety, QoL and change of JAK-2 allelic burden will be analyzed, using the secondary endpoints outlined, to provide a more comprehensive picture on ropeginterferon alfa-2b compared to HU in patients with PV.

CONTINUATION-PV

Primary objectives:

To assess the long-term efficacy of ropeginterferon alfa-2b or BAT in terms of disease response rate in patients diagnosed with PV, who were previously treated with ropeginterferon alfa-2b or HU in the PROUD-PV Study and who completed this study.

To assess the long-term efficacy including changes in disease burden present during PROUD-PV Study, in patients diagnosed with PV, who were previously treated with ropeginterferon alfa-2b or HU in the PROUD-PV Study. Disease burden is defined as disease-related signs (clinically significant splenomegaly) and disease-related symptoms (microvascular disturbances, pruritus, headache), assessed by the investigator.

Secondary objectives:

To further assess the long-term efficacy, safety, quality of life, and change of JAK2 allelic burden in patients ropeginterferon alfa-2b.

Additionally, for patients using the pre-filled 250 μg injection pen to collect additional information on self-administration of ropeginterferon alfa-2b.

### Supplemental Statistical Methods

The statistical methods have been published previously.^1^

Determination of sample size

The null hypothesis in PROUD-PV was inferiority of the response to ropeginterferon alfa-2b with a margin of at least 10.5% from the response for hydroxyurea after 12 months. This hypothesis was tested against the alternative at a one-sided significance level of 2.5% by estimating the two-sided 95% CI of the difference in response between the treatment groups and comparing the lower limit of the confidence interval to the non-inferiority margin. The noninferiority margin of 10.5% in PROUD-PV was derived by halving the assumed control effect of 25%, after subtracting 4% to allow for random fluctuations in response due to the nature of the disease ([25%– 4%]/2=10.5%).

Sample size in PROUD-PV was based on the expected response rates at 12 months (at least 25% overall: 32% for ropeginterferon alfa-2b and 12% for control treatment, allowing for a 20% drop-out rate). Thus, 252 patients (126 per arm) were required to detect the difference in response rate between treatment arms at 1% (two-sided) significance level with 90% power, using a standard chi-square test. Considering divisibility by eight strata, 128 patients per treatment group (256 in total) were planned to be enrolled. No formal hypothesis was planned to be tested in the CONTINUATION-PV Study; therefore, no power calculation or sample size recalculation was performed for the extension study.

Randomization and masking

Randomization was block-stratified by eight strata (previous hydroxyurea treatment [yes/no], age at screening [≤60 or >60 years] and history of thromboembolic events [yes/no]). Treatment assignment was conducted at the sites using an electronic case report form system (Merge eClinical OS). The randomization list was generated by the statistician using SAS version 9.3. Patients were defined as enrolled upon providing the investigator or delegate with their written informed consent.

Analyses performed at 60 months

Analyses were performed according to the CONTINUATION-PV statistical analysis plan (patients analyzed by treatment assigned; full analysis set) and included the rate of CHR without the spleen criterion according to modified ELN criteria^5^, *JAK2*V617F allele burden, molecular response according to ELN criteria^5^, and adverse events. Last observation carried forward was imputed for *JAK2*V617F allele burden and molecular response, and for a sensitivity analysis of CHR. Additionally, post-hoc analyses were conducted as follows. The number of patients in different allele burden categories at Month 60 was compared between the study arms using the Fisher test. The number of patients meeting all presumed criteria for treatment discontinuation (complete hematologic response [CHR] for ≥2 years, and no disease progression, thromboembolic events, or worsening of PV signs and symptoms during treatment) was compared using the Fisher test. In patients in the ropeginterferon alfa-2b arm with baseline *JAK2*V617F allele burden ≥10% who remained on study at Month 60 (thus were treated for ≥5 years) and achieved an allele burden of <10% at Month 60, univariate and multivariate logistic regression models were used to determine predictive baseline factors (demographics, presence of non-driver mutations, time since diagnosis, duration of any previous hydroxyurea treatment, hematocrit, leukocyte and platelet count, spleen size and presence of disease-related symptoms) for achievement of allele burden <10% at Month 60, and the association of allele burden <10% with other parameters (derived 4-weekly dose level, CHR, duration of maintained CHR, individual hematologic parameters, phlebotomy need, thromboembolic events, disease progression and quality of life [EQ-5D-3L score]) at Month 60 was determined using Fisher’s exact test or Wilcoxon’s test.

### Important changes to methods after trial commencement

The primary objective in PROUD-PV was originally to assess the superiority of ropeginterferon alfa-2b versus HU regarding disease response. Prior to database lock or sponsor unblinding, newly published data indicated that the hematological response among patients treated with HU may exceed the response rate assumed in PROUD-PV, which was estimated based on earlier literature.^6,7^ Therefore, the study objective in PROUD-PV was revised to the assessment of non-inferiority of ropeginterferon alfa-2b versus HU regarding disease response in a protocol amendment dated 15 June 2016.

All protocol amendments are described below.

There were three amendments to the PROUD-PV study protocol:

*Amendment 1*: According to FDA submission requirements, the endpoint "durable disease response" was added in the study protocol; however, for study submission in Europe the primary endpoint "disease response at 12 months" remained unchanged. A separate, USA‑specific statistical analysis plan (SAP) was written. All endpoints as per study protocol were included in both SAPs; the statistical methods and sample size justification were consistent in both SAPs. In addition, the power calculation for "durable disease response" was presented in the separate USA‑ specific SAP.

*Amendment 2*: Clarification on patients who were to have an immunogenicity sample drawn on week 4 and who had already started the study at the time this was implemented.

*Amendment 3*: The primary objective was changed from “To demonstrate superiority of ropeginterferon alfa-2b vs. hydroxyurea in terms of disease response rate in both hydroxyurea naïve and currently treated patients, diagnosed with Polycythemia Vera” to “To demonstrate noninferiority of ropeginterferon alfa-2b vs. hydroxyurea in terms of disease response rate in both hydroxyurea naïve and currently treated patients, diagnosed with Polycythemia Vera.”

There were five amendments to the CONTINUATION-PV study protocol:

*Amendment 1*: Implementation of the hydroxyurea arm to CONTINUATION-PV for collecting further data from the patients who were randomized into the hydroxyurea arm in PROUD-PV.

*Amendment 2*: Study endpoints were changed, and co-primary endpoints added. Rewording of hydroxyurea arm to best available treatment also included patients with other first line therapy than hydroxyurea. Efficacy parameters were collected for both arms every three months.

*Amendment 3:* A sub-study was introduced at selected sites to investigate tolerability of a 500 µg pen size in patients who would otherwise use two 250 µg pens*.*

*Amendment 4:* A pharmacokinetic sub-study was introduced. Specific assessments were only to be performed if clinically indicated and according to the investigator’s discretion.

*Amendment 5:* The study duration was extended by one year and the frequency of assessment visits was reduced from 3-monthly to 6-monthly*.*

## Supplementary results

### Dosing

Following initial up-titration, the ropeginterferon alfa-2b dose remained stable over time, with a median cumulative monthly dose of 499 μg (interquartile range [IQR]: 272‑898 μg) at the end of the fifth year. Most patients in the control arm (88% at Month 60) continued hydroxyurea treatment, for which the dose level was consistent over time, with a derived median daily dose of 1000 mg at Month 60 (IQR: 750-1,500 mg).

## Tables

### Patient characteristics

Supplementary Table 4: Demographic and baseline characteristics at screening in the PROUD-PV Study (Full Analysis Set)

|  |  | **Ropeginterferon alfa-2b** | | **Control** | | |
| --- | --- | --- | --- | --- | --- | --- |
| **Variable** | **Statistics** | **Patients enrolled in PROUD-PV** | **Patients enrolled in**  **CONTINUATION-PV** | **Patients enrolled in PROUD-PV** | **Patients enrolled in**  **CONTINUATION-PV** |  |
|  |  | **N = 127** | **N = 95** | **N = 127** | **N = 76** |  |
| Age [year] | Median (Q1-Q3) | 60.0 (52.0-66.0) | 58.0 (50.0-64.0) | 60.0 (48.0-67.0) | 59.0 (49.0-65.5) |  |
|  | Range | 30.0-85.0 | 30.0-85.0 | 21.0-81.0 | 32.0-79.0 |  |
| Sex | Female, n (%) | 68 (53.5%) | 48 (50.5%) | 67 (52.8%) | 40 (52.6%) |  |
|  | Male, n (%) | 59 (46.5%) | 47 (49.5%) | 60 (47.2%) | 36 (47.4%) |  |
| Race | White, n (%) | 127 (100.0%) | 95 (100.0%) | 127 (100.0%) | 76 (100.0%) |  |
| Body mass index (kg/m ^2^) | Median (Q1-Q3) | 25.9 (23.8-28.8) | 25.9 (24.0- 29.1) | 26.3 (24.2-29.0) | 26.3 (24.3-29.5) |  |
| Hematocrit [%] | Median (Q1-Q3) | 47.1 (44.2-51.3) | 47.7 (44.4-52.0) | 48.0 (44.6-52.5) | 49.9 (46.2-53.1) |  |
| Platelet count [10 ^9^/L] | Median (Q1-Q3) | 485.0 (350.0-671.0) | 488.0 (350.0-701.0) | 452.0 (329.0-666.0) | 451.0 (329.0-678.5) |  |
| Leukocyte count [10 ^9^/L] | Median (Q1-Q3) | 10.6 (8.0-13.4) | 10.9 (8.0-14.6) | 10.5 (7.9-14.5) | 11.3 (8.7-15.1) |  |
| Time since diagnosis of polycythemia vera (months) | Median (Q1-Q3) | 1.9 (0.7-11.2) | 1.8 (0.6-6.8) | 3.6 (0.7-20.0) | 1.6 (0.7-15.1) |  |
|  | Range | 0.0-145.5 | 0.0-145.5 | 0.0-126.3 | 0.0-91.6 |  |
| Presence of disease-related symptoms | Yes, n (%) | 23 (18.1%) | 15 (15.8%) | 26 (20.5%) | 17 (22.4%) |  |
| *JAK2*V617F allele burden (%) | n | 126 | 94 | 125 | 74 |  |
|  | Mean (±SD) | 41.9 (±23.49) | 42.8 (±23.40) | 42.8 (±24.14) | 42.9 (±23.01) |  |
|  | Median (Q1-Q3) | 37.3 (23.2-59.3) | 37.3 (24.8-62.7) | 37.4 (22.9-63.3) | 38.1 (21.6-63.0) |  |
|  | Range | 0.0-94.9 | 2.6-94.9 | 0.0-89.8 | 2.5-86.6 |  |
| Presence of clinically significant splenomegaly* | n | 127 | 95 | 127 | 76 |  |
|  | Yes, n (%) | 12 (9.4%) | 7 (7.4%) | 15 (11.8%) | 8 (10.5%) |  |
| Spleen size [cm], if yes | Median | 15.3 | 15.5 | 15.5 | 15.0 |  |
|  | Range | 10.5-19.5 | 10.5-19.5 | 9.0-23.5 | 9.0-17.5 |  |
| If pre-treated with hydroxyurea, duration of treatment (months) | n | 45 | 30 | 37 | 20 |  |
|  | Median (Q1-Q3) | 10.2 (2.1-21.3) | 9.5 (2.8-25.1) | 7.9 (2.7-19.2) | 8.2 (2.6-23.0) |  |
|  | Range | 0.9-34.5 | 0.9-30.9 | 1.0-36.4 | 1.0-36.4 |  |

*As assessed by the investigator

### JAK2V617F allele burden

Supplemental Table 5: *JAK2*V617F allele burden category (last observation carried forward) and complete hematologic response at Month 60 among patients with *JAK2*V617F allele burden ≥10% at baseline

|  | **Ropeginterferon alfa-2b** | **Control** | **p-value comparing treatment arms**  **(Fisher test)** |
| --- | --- | --- | --- |
| Patients enrolled | 95 | 76 |  |
| Patients with *JAK2*V617F <10% at baseline* | 3 | 4 |  |
| Patients with *JAK2*V617F ≥10% at baseline | 92 | 72 |  |
| Patients with *JAK2*V617F ≥1% to <10% | n = 32 / 92 (34.8%) | n = 9 / 72 (12.5%) | 0.0011 |
| Range of baseline *JAK2*V617F | 10 - 84% | 12 - 78% |  |
| Patients with CHR** / n | 19 / 32 | 3 / 9 |  |
| Patients with *JAK2*V617F ≥0.01% to <1% | n = 13 / 92 (14.1%) | n = 1 / 72 (1.4%) | 0.0037 |
| Range of baseline *JAK2*V617F | 10 - 75% | 47 - 47% |  |
| Patients with CHR / n | 10 / 13 | 1 / 1 |  |
| Patients with *JAK2*V617F ≤0.014% (undetectable***) | n = 5 / 92 (5.4%) | n = 0 (0%) | NA |
| Range of baseline *JAK2*V617F | 14 - 56% | - |  |
| Patients with CHR / n | 4 / 5 | - |  |
| Patients with *JAK2*V617F <1% | n = 18 / 92 (19.6%) | n = 1 / 72 (1.4%) | 0.0002 |
| Range of baseline *JAK2*V617F | 10 - 75% | 47 - 47% |  |
| Patients with CHR / n | 14 / 18 | 1 / 1 |  |
| **Patients with *JAK2*V617F <10%** | **n = 50 / 92 (54.3%)** | **n = 10 / 72 (13.9%)** | **<0.0001** |
| **Range of baseline *JAK2*V617F** | **10 - 84%** | **12 - 78%** |  |
| **Patients with CHR / n** | **33 / 50** | **4 / 10** |  |

*For the 1 patient in the ropeginterferon alfa-2b arm and the 2 patients in the control arm who had no available baseline *JAK2*V617F allele burden measurement, the earliest available result was imputed.

**Defined as for the main efficacy analysis; discontinued patients are considered non-responders

***Limit of detection of the Ipsogen® JAK2 MutaQuant® kit; QIAGEN GmbH: 0.061%; limit of background: 0.014%; values ≤0.014% are considered undetectable.

*Abbreviations: CHR=complete hematologic response. Baseline is defined as the last measured value before the first study drug administration in PROUD-PV study.*

### Association of JAK2V617F allele burden of <10% at 5 years with baseline factors and other parameters at Month 60

Predictive baseline factors for achieving *JAK2*V617F allele burden <10% at Month 60 were examined in patients in the ropeginterferon alfa-2b arm, but not the control arm, due to the low number of hydroxyurea/BAT-treated patients who reached this threshold. Lower age and lower *JAK2*V617F allele burden at baseline were found to be predictive factors for allele burden <10% using both univariate and multivariate logistic regression models (median age was 54.0 years among patients with allele burden <10% versus 63.0 years in patients with allele burden ≥10%; univariate and multivariate analyses p=0.005 and p=0.002; median allele burden was 36.8% versus 46.3%, p=0.03 and p=0.02 respectively) (Supplemental Table 7). Median platelet and leukocyte counts at baseline were lower among patients achieving allele burden <10% at Month 60 versus ≥10% (422.0 x 10^9^/L versus 498.0 x 10^9^/L and 10.3 x 10^9^/L versus 11.8 x 10^9^/L, respectively) but a significant correlation was only seen in the multivariate analysis (p=0.006 and p=0.04, respectively). The absence of non-driver mutations was predictive only in the univariate analysis (p=0.02). Time since diagnosis of PV, presence of disease-related symptoms, and spleen size were not found to be predictive for *JAK2*V617F allele burden <10%. Of note, in this early PV population, only a small number of patients presented with disease-related symptoms (15/95; 15.8%) or clinically significant splenomegaly (7/95; 7.4%) at baseline.

Supplemental Table 6: Baseline characteristics predictive for achieving a *JAK2*V617F allele burden of <10% (last observation carried forward) at Month 60 in the ropeginterferon alfa-2b treatment arm among patients with an allele burden ≥10% at baseline and who were treated for at least 5 years*

|  | | | **Allele burden at Month 60 (LOCF)** | | | **Univariate model^1^** | **Multivariate model^2^** |
| --- | --- | --- | --- | --- | --- | --- | --- |
| **Variable** | **Statistics** |  | | **<10%** | **≥10%** | **p-value** | **p-value** |
| Presence of other mutations | Missing |  | | 1 | 1 |  |  |
|  | No |  | | 34 (94.4%) | 20 (71.4%) |  |  |
|  | Yes |  | | 2 (5.6%) | 8 (28.6%) | 0.0224 | 0.5925 |
| Age (years) | n |  | | 37 | 29 |  |  |
|  | Mean (±SD) |  | | 52.3 (±8.69) | 59.5 (±9.97) | 0.0051 | 0.0019 |
|  | Median (Q1-Q3) |  | | 54.0 (46.0-60.0) | 63.0 (50.0-67.0) |  |  |
|  | Range |  | | 36.0-68.0 | 37.0-76.0 |  |  |
| Gender | Female |  | | 15 (40.5%) | 15 (51.7%) |  |  |
|  | Male |  | | 22 (59.5%) | 14 (48.3%) | 0.3662 | 0.4958 |
| BMI (kg/m^2^) | n |  | | 37 | 29 |  |  |
|  | Mean (±SD) |  | | 26.8 (±4.15) | 26.8 (±3.67) | 0.9316 | 0.2732 |
|  | Median (Q1-Q3) |  | | 27.0 (24.5-28.8) | 26.1 (24.0-29.3) |  |  |
|  | Range |  | | 17.2-39.5 | 21.6-34.9 |  |  |
| Time since diagnosis of polycythemia vera (months) | n |  | | 37 | 29 |  |  |
|  | Mean (±SD) |  | | 8.6 (±24.30) | 9.2 (±18.97) | 0.9079 | 0.5545 |
|  | Median (Q1-Q3) |  | | 1.4 (0.6-5.9) | 1.2 (0.5-6.8) |  |  |
|  | Range |  | | 0.0-145.5 | 0.1-88.4 |  |  |
| Duration of previous treatment with hydroxyurea (months) | n |  | | 37 | 28 |  |  |
|  | Mean (±SD) |  | | 3.4 (±8.12) | 3.2 (±7.77) | 0.9001 | 0.9267 |
|  | Median (Q1-Q3) |  | | 0.0 (0.0-1.9) | 0.0 (0.0-0.8) |  |  |
|  | Range |  | | 0.0-30.7 | 0.0-30.1 |  |  |
| *JAK2*V617F allele burden (%) | n |  | | 37 | 29 |  |  |
|  | Mean (±SD) |  | | 39.0 (±19.93) | 50.6 (±22.38) | 0.0342 | 0.0171 |
|  | Median (Q1-Q3) |  | | 36.8 (23.2-53.6) | 46.3 (30.6-67.8) |  |  |
|  | Range |  | | 10.0-83.9 | 16.2-92.0 |  |  |
| Hematocrit (%) | n |  | | 37 | 29 |  |  |
|  | Mean (±SD) |  | | 42.3 (±2.86) | 43.2 (±3.70) | 0.2609 | 0.3342 |
|  | Median (Q1-Q3) |  | | 42.1 (40.9-44.0) | 44.0 (41.0-44.8) |  |  |
|  | Range |  | | 34.7-48.3 | 35.0-50.9 |  |  |
| Platelet count (10^9^/L) | n |  | | 37 | 29 |  |  |
|  | Mean (±SD) |  | | 464.8 (±231.32) | 552.6 (±259.00) | 0.1528 | 0.0064 |
|  | Median (Q1-Q3) |  | | 422.0 (285.0-650.0) | 498.0 (401.0-640.0) |  |  |
|  | Range |  | | 165.0-957.0 | 115.0-1179.0 |  |  |
| Leukocyte count (10^9^/L) | n |  | | 37 | 29 |  |  |
|  | Mean (±SD) |  | | 11.2 (±5.12) | 12.3 (±4.82) | 0.3934 | 0.0363 |
|  | Median (Q1-Q3) |  | | 10.3 (7.9-12.9) | 11.8 (9.4-14.6) |  |  |
|  | Range |  | | 3.0-23.4 | 4.2-25.8 |  |  |
| Spleen size (cm) | n |  | | 37 | 29 |  |  |
|  | Mean (±SD) |  | | 13.5 (±3.26) | 14.4 (±3.21) | 0.2707 | 0.1259 |
|  | Median (Q1-Q3) |  | | 13.5 (11.0-15.0) | 14.0 (12.5-15.5) |  |  |
|  | Range |  | | 9.0-25.0 | 9.0-23.0 |  |  |
| Presence of disease-related symptoms | No |  | | 33 (89.2%) | 24 (82.8%) |  |  |
|  | Yes |  | | 4 (10.8%) | 5 (17.2%) | 0.4535 | 0.5109 |

* Patients with baseline *JAK2*V716F allele burden ≥10% were included in the analysis; missing baseline values were not imputed.

^1^ Logistic regression with a single explanatory variable

^2^ Logistic regression including all baseline characteristics in one model

*Abbreviations: LOCF=last observation carried forward; BMI= body mass index. Baseline is defined as the last measured value before the first study drug administration in PROUD-PV study.*

Evaluation of the association of allele burden <10% with other parameters at Month 60 in patients treated with ropeginterferon alfa-2b for ≥5 years showed a clear relationship between allele burden and hematologic responses (Supplemental Table 8). Among patients achieving *JAK2*V617F allele burden <10%, 32/37 (86.5%) also had CHR at Month 60 compared with 17/29 (58.6%) among patients with allele burden ≥10% (p=0.02). Moreover, the median duration of the last maintained CHR was significantly longer among patients with an allele burden <10% than with allele burden ≥10% (41.8 months [IQR: 29.9-50.5] versus 30.3 months [IQR: 14.7-42.0]; p=0.03). Among patients with allele burden <10%, at Month 60, all had platelet counts <400 x 10^9^/L and leukocyte counts <10 x 10^9^/L; however, 6 still required phlebotomy in the fifth year, 2 had mild splenomegaly (spleen size 14.9 cm and 13.4 cm) and 2 had PV-related symptoms. Dosing level (derived 4-weekly dose of <250 µg, 250-500 µg or >500 µg) had no apparent impact on the achievement of an allele burden <10% (p=0.9). The potential correlation between *JAK2*V617F allele burden and disease progression could not be assessed because only one case of progression to myelofibrosis was reported in the ropeginterferon alfa-2b arm. No significant correlation was found between occurrence of thromboembolic events and *JAK2*V617F allele burden <10% at Month 60 (p=0.5), but the low number of events (5 in total) does not allow firm conclusions to be drawn.

Supplemental Table 7: Association of *JAK2*V617F allele burden <10% (last observation carried forward) with other parameters at Month 60 among patients in the ropeginterferon alfa-2b treatment arm with a baseline allele burden ≥10% and who were treated for at least 5 years*

| **Variable** | **Statistics** | **Allele burden <10% at Month 60 (LOCF)** | **Allele burden ≥10% at Month 60 (LOCF)** | **p-value** |
| --- | --- | --- | --- | --- |
| Derived dose per 4 weeks | <250 µg | 2 (5.4%) | 2 (6.9%) | Fisher test: 0.9173 |
|  | 250-500 µg | 11 (29.7%) | 7 (24.1%) |  |
|  | >500 µg | 24 (64.9%) | 20 (69.0%) |  |
| CHR | Non-responder | 5 (13.5%) | 12 (41.4%) |  |
|  | Responder | 32 (86.5%) | 17 (58.6%) | Fisher test: 0.0216 |
| Duration of maintained CHR (months)^1^ | n | 37 | 29 |  |
|  | Mean (±SD) | 37.9 (±16.25) | 28.6 (±17.47) | Wilcoxon test: 0.0311 |
|  | Median (Q1-Q3) | 41.8 (29.9-50.5) | 30.3 (14.7-42.0) |  |
|  | Range | 0.0 -57.8 | 0.0-56.5 |  |
| Hematocrit <45% without phlebotomy | No | 5 (13.5%) | 10 (34.5%) |  |
|  | Yes | 32 (86.5%) | 19 (65.5%) | Fisher test: 0.0740 |
| Hematocrit | n | 37 | 29 |  |
|  | Mean (±SD) | 40.4 (±3.10) | 41.8 (±5.20) | Wilcoxon test: 0.1508 |
|  | Median (Q1-Q3) | 40.2 (38.0-42.3) | 41.6 (39.0-46.0) |  |
|  | Range | 35.0-47.0 | 29.0-51.0 |  |
| Platelet count <400 x 10^9^/L | No | - | 5 (17.2%) |  |
|  | Yes | 37 (100%) | 24 (82.8%) | Fisher test: 0.0133 |
| Platelet count | n | 37 | 29 |  |
|  | Mean (±SD) | 175.1 (±58.83) | 246.2 (±124.09) | Wilcoxon test: 0.0173 |
|  | Median (Q1-Q3) | 154.0 (134.0-195.0) | 223.0 (158.0-286.0) |  |
|  | Range | 107.0-319.0 | 84.0-604.0 |  |
| Leukocyte count <10 x 10^9^/L | Yes | 37 (100%) | 29 (100%) |  |
| Leukocyte count | n | 37 | 29 |  |
|  | Mean (±SD) | 4.3 (±1.17) | 5.5 (±1.45) | Wilcoxon test: 0.0004 |
|  | Median (Q1-Q3) | 3.9 (3.5-4.9) | 5.5 (4.4-6.2) |  |
|  | Range | 2.5-7.3 | 3.0-8.9 |  |
| Phlebotomy need in the 5th year | No | 31 (83.8%) | 21 (72.4%) |  |
|  | Yes | 6 (16.2%) | 8 (27.6%) | Fisher test: 0.3647 |
| Thromboembolic events | No | 35 (94.6%) | 29 (100%) |  |
|  | Yes | 2 (5.4%) | - | Fisher test: 0.4998 |
| Disease progression | No | 37 (100%) | 29 (100%) |  |
| EQ-5D-3L quality of life total score | 5 | 26 (70.3%) | 18 (62.1%) | Fisher test: 0.0732 |
|  | 6 | 6 (16.2%) | 5 (17.2%) |  |
|  | 7 | 1 (2.7%) | 6 (20.7%) |  |
|  | 8 | 2 (5.4%) | - |  |
|  | 9 | 2 (5.4%) | - |  |
| EQ-5D-3L quality of life total score | n | 37 | 29 |  |
|  | Mean (±SD) | 5.6 (±1.14) | 5.6 (±0.82) | Wilcoxon test: 0.5640 |
|  | Median (Q1-Q3) | 5.0 (5.0-6.0) | 5.0 (5.0-6.0) |  |
|  | Range | 5.0-9.0 | 5.0-7.0 |  |
| Spleen size (cm) | n | 37 | 29 |  |
|  | Mean (±SD) | 12.4 (±1.81) | 12.8 (±2.56) | Wilcoxon test: 0.8468 |
|  | Median (Q1-Q3) | 12.4 (11.6-13.2) | 12.4 (11.2-13.7) |  |
|  | Range | 8.8-16.3 | 8.7-20.0 |  |
| Clinically significant splenomegaly^2^ | No | 35 (94.6%) | 28 (96.6%) |  |
|  | Yes | 2 (5.4%) | 1 (3.4%) | Fisher test: 1.0000 |
| Disease-related symptoms present at Month 60 | No | 35 (94.6%) | 27 (93.1%) |  |
|  | Yes | 2 (5.4%) | 2 (6.9%) | Fisher test: 1.0000 |
| Worsening of PV-related signs and symptoms^3^ (whole study period) | No | 34 (91.9%) | 28 (96.6%) |  |
|  | Yes | 3 (8.1%) | 1 (3.4%) | Fisher test: 0.6248 |

* Patients with baseline *JAK2*V716F allele burden ≥10% were included in the analysis; missing baseline values were not imputed.

^1^ Duration of maintained CHR at Month 60 is defined as the length of the period immediately prior to the Month 60 assessment visit in which CHR was achieved consistently.

^2^ Clinically significant splenomegaly was assessed according to the investigator’s clinical judgement.

^3^ Worsening of PV-related signs (clinically significant splenomegaly as assessed by the investigator) and symptoms (microvascular disturbances, pruritus or headache), was defined as the development of a new sign or symptom that was not present in the medical history, or an increase in severity grade of a pre-existing sign or symptom during the study.

*Abbreviations: LOCF=last observation carried forward; CHR= complete hematologic response.*

*Baseline is defined as the last measured value before the first study drug administration in PROUD-PV study.*

## Figures

### Patient disposition

Supplemental Figure 2: CONSORT flow diagram 

Analysed (n=127)

- Excluded from analysis (n=0)

Analysed (n=127)

- Excluded from analysis (n=0)

**Analysis**

Discontinued control (n=19)

- Administrative reasons (n=1)
- Adverse events (n=3)
- Withdrawal of consent (n=8)
- Lack of efficacy (n=2)
- Other (n=5)
- Lost to follow-up (n=0)

Allocated to control (n=130)

- Received allocated intervention (n=127)
- Did not receive allocated intervention (n=3)
- Withdrawal of consent (n=3)

Completed PROUD-PV (n=111)

Completed PROUD-PV (n=106)

Discontinued ropeginterferon alfa-2b (n=21)

- Administrative reasons (n=4)
- Adverse events (n=11)
- Withdrawal of consent (n=6)
- Lost to follow-up (n=0)

Allocated to ropeginterferon alfa-2b (n=127)

- Received allocated intervention (n=127)
- Did not receive allocated intervention (n=0)

**Follow-Up**

**Allocation**

**Enrolment**

Randomised (n=257)

Excluded (n=49)

- Not meeting inclusion criteria (n=39)
- Declined to participate (n=8)

Assessed for eligibility (n=306)

Did not roll over (n=35)

Did not roll over (n=11)

**Follow-Up**

**Allocation**

**Enrolment**

Enrolled in CONTINUATION-PV (n=76)

Continued with ropeginterferon alfa-2b (n=95)

- Received allocated intervention (n=95)
- Did not receive allocated intervention (n=0)

Enrolled in CONTINUATION-PV (n=95)

Started with best available therapy (n=76)

- Received allocated intervention (n=76)
- Did not receive allocated intervention (n=0)

Discontinued ropeginterferon alfa-2b (n=25)

- Administrative reasons (n=2)
- Adverse events (n=11)
- Withdrawal of consent (n=3)
- Lack of efficacy (n=3)
- Other (n=5)
- Lost to follow-up (n=1)

Discontinued control (n=19)

- Adverse events (n=2)
- Withdrawal of consent (n=8)
- Other (n=8)
- Lost to follow-up (n=1)

Analysed (n=76)

- Excluded from analysis (n=0)

Analysed (n=95)

- Excluded from analysis (n=0)

**Analysis**

## Members of the PROUD-PV Study Group

| **Principal Investigator** | **Affiliation** |
| --- | --- |
| Heinz Gisslinger | Medical University Vienna, Department of Internal Medicine I, Clinical Division of Hematology and Hemostaseology, Vienna, Austria |
| Ella Willenbacher | Medical University Innsbruck, Department of Internal Medicine V (Hematology and Oncology), Innsbruck, Austria |
| Richard Greil | Salzburg Regional Hospital, University Department of Internal Medicine III, Salzburg, Austria |
| Ernst Forjan | Hanusch Hospital, Department of Internal Medicine III, Vienna, Austria |
| Veronika Buxhofer-Ausch | Internal Medicine I for Hematology with Stem Cell Transplantation, Hemostasis and Medical Oncology, Ordensklinikum Linz Elisabethinen, Linz, Austria |
| Franz Bauer | University Hospital Graz, University Clinic of Internal Medicine, Clinical Department of Hematology, Graz, Austria |
| Liana Gercheva-Kyuchukova | Multiprofile Hospital for Active Treatment "Sveta Marina", Varna, Bulgaria |
| Georgi Mihaylov | Specialized Hospital for Active Treatment of Hematological Diseases, Clinic of Clinical Hematology, Sofia, Bulgaria |
| Vera Stoeva | Specialized Hospital for Active Treatment of Hematological Diseases, Clinic of Clinical Hematology, Sofia, Bulgaria |
| Pencho Georgiev | University Multiprofile Hospital for Active Treatment "Sveti Georgi", Clinic of Hematology, Plovdiv, Bulgaria |
| Liliya Sivcheva | Multiprofile Hospital for Active Treatment - Hristo Botev, First Department of Internal Medicine Vratsa, Bulgaria |
| Jiri Schwarz | Institute of Hematology and Blood Transfusion, Prague, Czech Republic |
| Jiri Mayer | University Hospital Brno, Clinic of Internal Medicine - Hematology and Oncology, Brno, Czech Republic |
| Petr Dulicek | University Hospital Hradec Kralove, Department of Clinical Hematology, Hradec Kralove, Czech Republic |
| Olga Cerna | University Hospital Kralovske Vinohrady, Clinic of Internal Hematology, Prague, Czech Republic |
| Jean-Jacques Kiladjian | Université de Paris, CIC 1427, Inserm, Paris, France, Centre d’Investigations Cliniques, AP-HP, Hôpital Saint-Louis, Paris, France |
| Lydia Roy | University of Poitiers, INSERM, CHU Poitiers, Clinical Investigation Centre CIC1402, Department of Onco-Hematology, Poitiers, France |
| Mathieu Puyade | University of Poitiers, INSERM, CHU Poitiers, Clinical Investigation Centre CIC1402, Department of Onco-Hematology, Poitiers, France |
| Emilie Cayssials-Caylus | University of Poitiers, INSERM, CHU Poitiers, Clinical Investigation Centre CIC1402, Department of Onco-Hematology, Poitiers, France |
| Jose Miguel Torregrosa-Diaz | University of Poitiers, INSERM, CHU Poitiers, Clinical Investigation Centre CIC1402, Department of Onco-Hematology, Poitiers, France |
| Jerome Rey | Paoli-Calmettes Institute, Marseille, France |
| Dominik Wolf | University Hospital Bonn, Centre for Internal Medicine, Department of Internal Medicine III - Oncology, Hematology and Rheumatology, Bonn, Nordrhein-Westfalen, Germany |
| Steffen Koschmieder | Department of Hematology, Oncology, Hemostaseology, and Stem Cell Transplantation, Faculty of Medicine, RWTH Aachen University, Aachen, Nordrhein-Westfalen, Germany |
| Uwe Platzbecker | University Hospital Carl Gustav Carus, Medical Clinic and Polyclinic I, Dresden, Germany |
| Miklos Egyed | Kaposi Mor Teaching Hospital, Department of Internal Medicine II, Kaposvar, Somogy, Hungary |
| Tamas Masszi | St Istvan and St Laszlo Hospital of Budapest, Department of Hematology and Stem Cell Transplantation , Budapest, Hungary |
| Arpad Illes | University of Debrecen, Faculty of Medicine, Department of Hematology, Debrecen, Hungary |
| Zita Borbenyi | University of Szeged, Albert Szent-Gyorgyi Clinical Centre, Department of Internal Medicine II and Cardiology Centre, Hematology, Szeged, Hungary |
| Janos Jakucs | Bekes County Pandy Kalman Hospital, 1st Department of Medicine, Gyula, Hungary |
| Mario Cazzola | Foundation IRCCS Policlinico San Matteo, Pavia, Italy |
| Jolanta Starzak-Gwozdz | Frederic Chopin Provincial Teaching Hospital No. 1 in Rzeszow, Department of Hematology, Rzeszow, Poland |
| Krzysztof Warzocha | Institute of Hematology and Transfusion Medicine, Teaching Department of Hematology, Warsaw, Poland |
| Malgorzata Calbecka | Nicolaus Copernicus Municipal Specialist Hospital, Department of Hematology, Torun, Poland |
| Maria Soroka-Wojtaszko | Independent Public Teaching Hospital No.1 in Lublin, Department of Hematology-Oncology, Bone Marrow Transplantation and Chemotherapy, Lublin, Poland |
| Dorota Krochmalczyk | University Hospital in Krakow, Teaching Unit of the Hematology Department, Krakow, Poland |
| Aleksander Skotnicki | University Hospital in Krakow, Teaching Unit of the Hematology Department, Krakow, Poland |
| Nicoleta Berbec | Coltea Clinical Hospital, Bucharest, Romania |
| Horia Bumbea | Bucharest University Emergency Hospital, Bucharest, Romania |
| Andrei Cucuianu | "Prof. Dr. Ion Chiricuta" Institute of Oncology, Hematology Department, Cluj-Napoca, Romania |
| Delia Monica Dima | "Prof. Dr. Ion Chiricuta" Institute of Oncology, Hematology Department, Cluj-Napoca, Romania |
| Emanuil Gheorghita | Rapid Diagnostic Polyclinic SRL. Brasov, Romania |
| Mihaela Lazaroiu | Rapid Diagnostic Polyclinic SRL, Brasov, Romania |
| Alexander Myasnikov | Baranov Republican Hospital, Petrozavodsk, Republic of Karelia, Russian Federation |
| Irina Sokolova | Komi Republican Oncology Centre, Syktyvkar, Russian Federation |
| Elena Volodicheva | Tula Regional Clinical Hospital, Tula, Russian Federation |
| Viktor Rossiev | V.D. Seredavin Samara Regional Clinical Hospital Samara, Russian Federation |
| Vera Yablokova | Yaroslavl Regional Clinical Hospital, Department of Hematology, Yaroslavl, Russian Federation |
| Anna Vallova | University Hospital with Outpatient Clinic F.D. Roosevelt Banska Bystrica, Banska Bystrica, Slovakia |
| Antonia Hatalova | University Hospital Saint Cyril and Metod Bratislava, Bratislava, Slovakia |
| Mikulas Hrubisko | University Hospital Saint Cyril and Metod Bratislava, Bratislava, Slovakia |
| Carlos Besses Raebel | Hospital del Mar, Department of Hematology, Barcelona, Spain |
| Halyna Pylypenko | Cherkasy Regional Oncology Centre, Department of Clinical Trials, Cherkasy, Ukraine |
| Polina Kaplan | Dnipropetrovsk City Multispecialty Clinical Hospital #4, Dnipro, Ukraine |
| Zvenyslava Masliak | Institute of Blood Pathology and Transfusion Medicine, Department of Hematology, Lviv, Ukraine |
| Sergiy Klymenko | National Research Centre for Radiation Medicine Institute of Clinical Radiology, Department of Hematology and Transplantation, Kyiv, Ukraine |
| Tamila Lysa | O.F. Herbachevskyi Regional Clinical Hospital, Hematology Centre, Zhytomyrv, Ukraine |

## Institutional review board/ independent ethics committee approval

The study protocols and subsequent amendments were submitted for approval to an Institutional review board (IRB) or independent ethics committee (IEC). The study protocol, a sample of the patient information and ICF, any other materials provided to the patients, and any further requested information was submitted. The study approval letter was required before any patient was exposed to a study-related procedure (i.e. prior to enrollment).

The approving central ethics committees in each country were as follows. **Austria**: Ethikkommission der Medizinische Universität Wien (Ethics Committee of Vienna Medical University), Borschkegasse 8b/6, 1090 Vienna; **Bulgaria**: Ethics Committee for Clinical Trials, 8, Damyan Gruev St. 1303 Sofia; **Czech Republic**: Ethics Committee UH Brno, Jihlavska 20, 625 00 Brno; **France**: Comité de Protection des Personnes Ile-de-France IV, Hôpital Saint-Louis, Porte 5 du carré historique; 1er étage Centre de Formation, 1 avenue Claude Vellefaux, 75475 Paris Cedex 10; **Germany**: Ethik-Kommission an der medizinischen Fakultät der RWTH Aachen, Pauwelsstr. 30, 52074 Aachen; **Hungary**: Research Council Ethics Committee for Clinical Pharmacology, Alkotmány u. 25, 1051 Budapest; **Italy**: San Matteo Polyclinic Research Hospital (IRCCS) Foundation Scientific Institute for Research, Hospitalisation and Health Care of the Public sector, Viale Golgi, 19 – 27100 Pavia; **Poland**: Ethics Committee of Kujawsko-Pomorska, District Medical Chamber in Torun, 87-100 Toruń, ul. Chopina 20; **Romania**: Academy of Medical Sciences National Bioethics Committee for Medicines and Medical Devices, 19-21 Stefan cel Mare Av., Sector 2, 020125 Bucharest; **Russian Federation**: Ministry of Healthcare of the Russian Federation; Ethics Council, 3 Rakhmanovskiy per., 127994 Moscow; **Slovakia**: Ethics Committee of NsP F. D. Roosevelta (UH with Outpatient Clinic F. D. Roosevelt), Nám. L. Svobodu 1, 975 17 Banská Bystrica; **Spain**: CEIm Parc de Salut Mar C/Dr. Aiguader, 88, Edificio PRBB, 08003 Barcelona. There was no central ethics committee in **Ukraine**; the local approving ethics committees were: Local Ethics Committee within the Communal Nonprofit Enterprise "Cherkasy Regional Oncology Center of Cherkasy Regional Council". 18009, Cherkasy, 7 Mendelieieva Vul.; Ethics Committee within the Public Non-Profit Enterprise "City Clinical Hospital #4" under Dnipro City Council. 49102, Dnipro, 31 Blyzhnia Vul.; Ethics Committee (Bioethics and Deontology Committee) within State Institution "Institute of Blood Pathology and Transfusion Medicine under the National Academy of Medical Sciences of Ukraine". 79044, Lviv, 45 Henerala Chuprynky Vul.; Ethics Committee within the State Institution: National Research Center for Radiation Medicine under the Ukrainian National Academy of Medical Sciences. 119/121 Peremohy Prosp., 04050; Ethics Committee within Public Non-Profit Enterprise "O.F. Herbachevskyi Regional Clinical Hospital" under Zhytomyr Regional Council, 3 Chervonoho Khresta vul., Zhytomyr,10002.

## References

1. Gisslinger H, Klade C, Georgiev P, et al. Ropeginterferon alfa-2b versus standard therapy for polycythaemia vera (PROUD-PV and CONTINUATION-PV): a randomised, non-inferiority, phase 3 trial and its extension study. *Lancet Haematol*. 2020;7(3):e196-e208.

2. Barbui T, Barosi G, Birgegard G, et al. Philadelphia-negative classical myeloproliferative neoplasms: critical concepts and management recommendations from European LeukemiaNet. *J Clin Oncol*. 2011;29(6):761-770.

3. Barosi G, Birgegard G, Finazzi G, et al. A unified definition of clinical resistance and intolerance to hydroxycarbamide in polycythaemia vera and primary myelofibrosis: results of a European LeukemiaNet (ELN) consensus process. *Br J Haematol*. 2010;148(6):961-963.

4. Michiels JJ, Berneman ZN, Schroyens W, Van Vliet HH. Pathophysiology and treatment of platelet-mediated microvascular disturbances, major thrombosis and bleeding complications in essential thrombocythaemia and polycythaemia vera. *Platelets*. 2004;15(2):67-84.

5. Barosi G, Birgegard G, Finazzi G, et al. Response criteria for essential thrombocythemia and polycythemia vera: result of a European LeukemiaNet consensus conference. *Blood*. 2009;113(20):4829-4833.

6. Alvarez-Larrán A, Angona A, Ancochea A, et al. Masked polycythaemia vera: presenting features, response to treatment and clinical outcomes. *Eur J Haematol*. 2016;96(1):83-89.

7. Alvarez-Larrán A, Pereira A, Cervantes F, et al. Assessment and prognostic value of the European LeukemiaNet criteria for clinicohematologic response, resistance, and intolerance to hydroxyurea in polycythemia vera. *Blood*. 2012;119(6):1363-1369.
